# Nociceptive and Neuronal Evaluation of the Sciatic Nerve of Wistar Rats Subjected to Compression Injury and Treated with Resistive Exercise

**DOI:** 10.1155/2016/6487160

**Published:** 2016-08-10

**Authors:** Juliana Sobral Antunes, Keli Lovison, Jhenifer Karvat, Ana Luiza Peretti, Lizyana Vieira, Guilherme Hideaki Higuchi, Rose Meire Costa Brancalhão, Lucinéia de Fátima Chasko Ribeiro, Gladson Ricardo Flor Bertolini

**Affiliations:** State University of Western Paraná (UNIOESTE), Universitaria Street, 2069, P.O. Box 711, 85819-110 Cascavel, PR, Brazil

## Abstract

*Background*. To investigate the climb stairs resistance exercise on nociception and axonal regeneration in the sciatic nerve of rats.* Methods*. 24 Wistar rats were divided: control group (CG—no injury), exercise group (EG—no injury with physical exercise), lesion group (LG—injury, but without exercise), and treated group (LEG—injury and physical exercise). LG and LEG were subjected to sciatic nerve compression with hemostat. From the 3rd day after injury began treatment with exercise, and after 22 days occurs the removal of a nerve fragment for morphological analysis.* Results*. Regarding allodynia, CG obtained values less than EG (*p* = 0.012) and larger than LG and LEG (*p* < 0.001). Histological results showed that CG and EG had normal appearance, as LG and LEG showed up with large amounts of inflammatory infiltration, degeneration and disruption of nerve fibers, and reduction of the myelin sheath; however LEG presented some regenerated fibers. From the morphometric data there were significant differences, for nerve fiber diameter, comparing CG with LG and LEG and comparing axon diameter and the thickness of the myelin of the CG to others.* Conclusion*. Climb stairs resistance exercise was not effective to speed up the regenerative process of axons.

## 1. Introduction

Peripheral nerve injuries represent a clinical problem and cause a profound and permanent impact on the patients' life quality by altering their ability to perform activities of daily living, as well as their return to work [1]. It is estimated that around twenty million Americans suffer peripheral nerve injuries, resulting in approximately $150 billion spent each year on health care in the United States [2].

The most common causes of peripheral nerve injuries are motor vehicle accidents, lacerations with sharp objects, fractures, and firearm or weapon injuries, among others [3]. In a retrospective study, Saadat et al. [4] evaluated 16,753 patients with some form of peripheral nerve injury and found that 90% of these were under 50 years of age, and the main causes that led to this problem were accidents transits to 58% of respondents and falls to 25%, demonstrating that the traumatic causes are commonly responsible for this type of injury, which mainly occur in individuals with productive age.

In cases of peripheral nerve lesions in the lower limbs, the most commonly affected nerve is the sciatic [3], and injuries occur mainly due to nerve root compression, femoral neck fracture, hip dislocation, contusions, and iatrogenic causes, which can generate motor and sensory deficits and neuropathic pain along the nerve pathway [5].

Although the peripheral nervous system (PNS) has an intrinsic ability to regenerate, the treatment options available, in compression severe cases, produce unsatisfactory results, and currently the treatment of choice is microsurgical repair [1]; however, special attention has been given to the development of nonsurgical therapeutic approaches to aid in the regeneration process and functional improvement after peripheral nerve injury, among which stands out the exercise [6].

The exercise can contribute to the peripheral nerve injury treatment, at least in part, because it induces the synthesis of neurotrophic factors, which stimulate nerve growth. Therefore, therapeutic exercise could form a useful means for the stimulation of growth and regeneration of peripheral axons [7]. Some neurotrophic factors are produced and released by Schwann cells and then cause a neurotropism effect, that is, guiding the nerve stump to its target organ. Different factors have been identified including nerve growth factor (NGF) and brain derived neurotrophic factor (BDNF) [8].

Furthermore, the literature shows that physical exercise could alleviate neuropathic pain, which may cause the release of endogenous opioids in the body, which consequently leads to reduced pain [9]. Then the physical exercise could influence the treatment of peripheral nerve injuries. However, most of the studies that made use of exercise for this purpose used aerobic exercise, and few studies have used resistive exercise [10]. Thus, the aim of this study was to investigate the influence of resistance exercise on nociception and axonal regeneration in the sciatic nerve of Wistar rats.

## 2. Materials and Methods

This study is characterized as an experimental research, with quantitative and random nature, which was developed after approval by the Ethics Committee on Animal Use (CEUA) of Universidade Estadual do Oeste do Paraná (Unioeste).

### 2.1. Groups

The sample consisted of 24 Wistar rats, males, mean age 10 weeks, weighing on average 352 g (375 g after the experiment), kept in standard boxes of polypropylene, in environment of 23 ± 1°C temperature, with photoperiod of 12 hours, and receiving food and water ad libitum.

The animals were randomized into four groups consisting of six animals each.

- Control group (CG): the animals were not injured or submitted to the physical exercise protocol.
- Exercise group (GE): the animals were uninjured and perform resistance physical exercise protocol.
- Injury group (LG): animals were injured.
- Lesion exercise group (LEG): animals were injured and underwent resistance physical exercise.

### 2.2. Lesion Protocol

The animals were subjected to sciatic nerve constriction injury, previously weighed, and anesthetized with ketamine (95 mg/kg) and xylazine (12 mg/kg) intraperitoneally. After verification of the animal's state of consciousness, through the clamping of the tail and interdigital folds, trichotomy of the right posterior thigh and disinfecting the area with the use of povidone were held. Then, an incision parallel to the biceps femoris fibers to expose the sciatic nerve and subsequent compression of it was taken with a hemostat for 30 seconds in order to reproduce chronic pain on nerve path [11].

The clamping pressure was standardized for all animals, using as reference the second tooth rack hemostat, being performed by the same researcher. After the clamping, a tag at the lesion site was performed by means of graft suture using nylon filament 10.0 in order to facilitate the location of the injured nerve collecting region [12]. Finally, the suture plans and povidone applied over the incision were made, and then the animals were housed in the same presurgical conditions.

### 2.3. Exercise Protocol

To carry out the treatment with resistance exercise, a vertical wooden ladder was used, which has 67 steps of iron, height of 1.18 m, width of 20.5 cm, and tilt 60°. At the top of the stairs, a dark box with a height of 18.5 cm and width of 15 cm, in which the animals were resting between one exercise series and another, was placed [13].

The procedure consists in the following: the animal performs two sets of 10 climbs of the ladder with overhead 100 grams adapted to its tail and with one-minute interval between one series and another. Treatment with climb stairs resistance exercise started from the third day after surgery (3rd PS—postsurgery) and they were performed 5 days per week, during 3 weeks, and they had 2 days break every 5 days. To perform the exercise on the stairs, all the animals were previously acquainted with the equipment for two weeks prior to surgery.

### 2.4. Allodynia Evaluation

The method used to analyze and to measure allodynia was the paw withdrawal threshold with the aid of a digital analgesy-meter, filament type Von Frey. The equipment consists of an arm with a disposable polypropylene tip, with the ability to measure between 0.1 and 1000 grams, and an amplifier connected to box, that is, a pressure transducer adapted to a digital counterforce expressed in grams [14].

The filament was applied to the plantar region of the right hindlimb paw. Therefore, the animal was kept in high wooden box, with screen floor and the filament positioned at 90 degrees with the animal's paw; pressure was applied with gradual increase in this region, until the animal withdraws its limb, by noting then the value of the withdrawal threshold indicated by the apparatus. To facilitate the adaptation of animals to this instrument in the three days prior to the injury, the simulation was carried out for this evaluation [15].

The evaluations related to allodynia occurred at the following times: a first assessment of baseline latency (BL) was before injury; the second evaluation (EV2) was on the 3rd PS before resistance exercise training (RET) and EV3 was on the same day, immediately after RET; the EV4 was on postoperative day 7 after RET; EV5 was on the 10th PS before RET; EV6 was on the 14th PO after RET; EV7 was on 21st PO after the last RET; and the last evaluation (no resistance physical exercise), EV8, occurred in 22nd PO, prior to euthanasia of animals.

### 2.5. Euthanasia

In the end of the 22nd PS, the animals were weighed and again anesthetized with ketamine and xylazine, and 2 cm fragments were dissected from the right sciatic nerve distal. This had been compressed to carry out the morphological and molecular analyses. Then, the animals were euthanized by decapitation.

### 2.6. Morphological Analysis

A part of the collected nerve fragments was set at bouin, embedded in paraffin, and subjected to cross sections of 5 *μ*m in thickness, with subsequent staining with hematoxylin and eosin (HE) to perform the histological analysis. Another portion of these fragments was fixed in paraformaldehyde 4% and postfixed in osmium tetroxide, processed for histological technique routine, and subjected to cross sections of 5 *μ*m, for performing the morphometric analysis [16].

In histology analysis, the features shown on the slide collection were observed and described, as the following structures: epineurium, perineurium and endoneurium, nerve fiber, inflammatory infiltrate, Schwann cells, fibroblasts, and blood vessels. The results were presented descriptively, from a detailed observation of the slide collection and illustrated in a histological board mounted with photomicrograph images of each group.

### 2.7. Morphometric Analysis

From the histological slides prepared, a section of each was selected which was photographed by Olympus BX 50 device. Using 100x objective, images were captured in 4 visual fields by systematically being located in the upper left quadrant, upper right, lower right, and bottom left, following the recommendations of Geuna et al. [17], and later images were analyzed using Image Pro-Plus 6.0.

Based on the photomicrographs images were performed to measure the diameter of the nerve fiber (DNF), the axon (DAX), the thickness of the myelin sheath (TMS), and the ratio G (the ratio of the DAX/DNF). These measurements were made at 100 axons by nerve, 25 in each quadrant in order to obtain an equivalent number for comparison.

### 2.8. Statistical Analysis

The results were expressed using descriptive statistics. The normality of the data was analyzed using the Shapiro-Wilk test. Then the results of functional evaluation were submitted to inferential statistics by ANOVA mixed measures with Bonferroni posttest; the results of morphometric analysis by one-way ANOVA with post-*t*-test and the results of molecular analysis were submitted to Kruskal-Wallis test. In all cases, the significance level was of *α* = 5%.

## 3. Results

### 3.1. Allodynia Assessment

Analyzing the data of the eight evaluations, a significant difference (*F* (7; 15.01) = 20.55, *p* < 0.001) in the comparison between groups and between assessments was observed.

Comparing the groups, there was significant difference between CG and the others, in which it showed lower results than EG (*p* < 0.012) and higher than LG (*p* < 0.001) and LEG (*p* < 0.001). Also significant was the comparison EG with LG and LEG, wherein the EG results were higher (*p* < 0.001). For comparisons between evaluations, it was observed that BL was higher than EV2–EV7 (3rd to 21st PS) (*p* < 0.001) and greater than EV8 (22nd PO) (*p* = 0.001), and EV4 (7th PS) was lower compared to EV7 (21st PS) (*p* = 0.002) and EV8 (22nd PS) (*p* = 0.011) (Table 1).

### 3.2. Histological Evaluation

**Figure 1:**
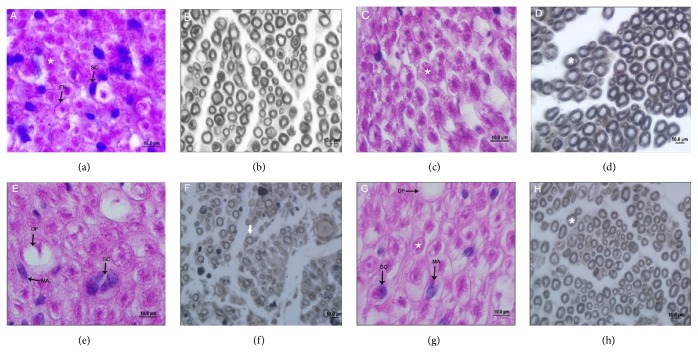
Photomicrographs of the sciatic nerve of Wistar rats in cross section, and the 1st column images are stained with hematoxylin and eosin and the 2nd column with osmium tetroxide. ((a) and (b)) Control group of nerve fibers intact (*∗*), surrounded by endoneurium (EN) and the presence of Schwann cell nuclei (SC). In (c) and (d), exercise group, notice the same characteristics of the control group. In (e) and (f), injury group, note the presence of several degenerated nerve fibers (DF), inflammatory infiltration with macrophages (MA), and giant cell clumps (GC), where (f) displayed thin myelin sheath (thick white arrow). In (g) and (h) group injury and exercise, there were degenerated nerve fibers (DF), core Schwann cell (SC), and inflammatory infiltrate (MA) but a large number of fibers with normal appearance (*∗*).

Histological analysis of the sciatic nerve CG revealed that the nerve fibers were of normal range, with different diameters, and revealed the presence of Schwann cell nuclei at the periphery of the myelin sheath and fibroblast nuclei in the endoneurium. Perineurium consists of connective tissue modeled; it performed involving the whole nerve, forming concentric layers around the same; fibroblast nuclei were also visualized. Adjacent to the perineurium was observed epineurium which also consists of connective tissue but not modeled (Figures 1(a) and 1(b)).

In the EG, the morphological approaches to what was observed in the control group are that the nerve fibers are presented in an organized manner, and no inflammatory cells or nerve fiber degeneration was found (Figures 1(c) and 1(d)).

However, at the LG, there was disruption of the nervous tissue with nerve fiber degeneration, increasing the number of Schwann cell nuclei, large amount of inflammatory infiltrate, with the presence of macrophages, forming giant cell clumps, increase in the amount of fibroblasts, and nerve fibers of smaller diameter compared to the control group. Additionally, the myelin sheath is made thinner and in some cases absent (Figures 1(e) and 1(f)).

In the LEG, very heterogeneous nerve fibers were noted, with being morphologically similar to the injury group, but other nerve fibers presented similar to that displayed in the control group, progressing to degree of normality (Figures 1(g) and 1(h)).

### 3.3. Morphometric Evaluation

**Figure 2:**
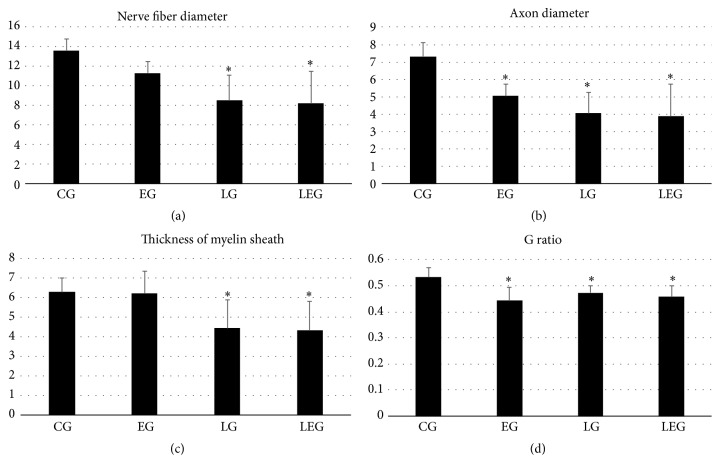
Graphical representation of results for morphometry. (a) Nerve fiber diameter, which is smaller in injured groups; (b) axon diameter, which was lower in the group only exercised and injured; (c) thickness of the myelin sheath, which was also lower in lesioned groups; and (d) G ratio, which was higher in the control group compared to the others. ^*∗*^Significant difference considering *p* < 0.05.

Regarding the nerve fibers diameter yielded significant differences (*F* (3; 20) = 7.69, *p* = 0.0016), when compared to the CG with the LG and LEG, and the value of *p* < 0.001 for both. Also when comparing EG with LG and LEG, *p* = 0.0439 and *p* = 0.0273, respectively, and the GC and EG values are greater (Figure 2(a)). We noticed smaller diameters in just injured groups.

The diameter of the axon yielded significant differences (*F* (3; 20) = 10.27, *p* = 0.0004), when compared to the CG with the other groups, with values of *p* = 0.004 compared to EG and for the other *p* < 0.001, and the DAX was higher in the control group (Figure 2(b)), whereas, in this variable, the injured group and the resistance physical exercise group evidenced lower values than the control group.

As regards the thickness of the myelin sheath, the result was similar to nerve fiber diameter (*F* (3; 21) = 5.49, *p* = 0.0062); that is, CG and EG had significant differences compared to LG and LEG. Then, by comparing the CG with LG, there was *p* = 0.0189; CG with LEG presented *p* = 0.0045; EG with LG obtained value of *p* = 0.0217; and finally EG with LEG resulted in *p* = 0.0053. That is, the thickness of the myelin sheath was higher in the control group and the group ladder in relation to the injury and the treated group (Figure 2(c)).

About the G ratio the results were significant (*F* (3; 20) = 6.10, *p* = 0.0043), when comparing the CG with EG, LG, and LEG and the values of *p* < 0.001, *p* = 0.0161, and *p* = 0.0033, respectively (Figure 2(d)), and the injured group and the resistance physical exercise group evidenced lower values than the control group.

## 4. Discussion

About allodynia results in the plantar region of the animals right hindlimb, it was observed that, with the passing of the evaluations, the values are not matching the assessment before injury, which is in all cases greater, indicating that the exercise was not effective to increase the nociceptive threshold. However, only EV4 was significantly smaller than the other two. This indicates that, with the passage of time, there was a small increase in the nociceptive threshold.

The study by Galdino et al. [18] investigated the influence of different protocols of resistance exercises with the use of a weight lifting model on the nociceptive threshold of healthy Wistar rats, and 2 protocols were used: the first was the animal performing 15 sets of 15 repetitions of the exercise and the second protocol was 3 sets of 10 repetitions, both with load of up to 75% of one repetition maximum for 12 weeks. They concluded that the exercise with greater intensity managed to increase the nociceptive threshold of the animals. This may be an explanation for the lack of results in the nociceptive threshold for the group that conducted the injury associated with exercise, in this study, because the applied exercise intensity may have been too light.

Checking the comparison between the groups it was observed that the nonlesioned group showed significantly superior results to injured. However, the control group achieved a lower result than did only exercise, showing that although the exercise was not significantly effective in promoting increased nociceptive threshold, a sedentary lifestyle, represented in CG, it is not beneficial, since, in EG, the exercise promoted increased nociceptive threshold. That is, the climb stairs resistance exercise increased the nociceptive threshold in healthy animals but was not enough to influence the degree of neuropathic pain in the group of injured and treated animals.

Corroborating these findings, Mazzardo-Martins et al. [19] studied the hyponociceptive effect of high-intensity swimming exercise in a chemical model of nociception and the mechanisms involved in this effect in mice and compared with the control group and a group that was associated with the exercise of the use of naloxone. Observe that the swimming exercise produced hyponociceptive effects, by means of a greater release of endogenous opioids, again confirming that high-intensity exercise is possibly the most effective for pain studies.

Another factor to be highlighted is the time it took for the animals to be reevaluated after the exercise, which may have influenced the results of this study. According Galdino et al. [20], who evaluated the influence of resistance exercise weightlifting in Wistar rats, 15 sets of 15 repetitions with a load of 70% of 1RM on the antinociception, and noted that the exercise was effective, however, its effect was not lasting. The nociceptive threshold stimulated by pressure increased immediately after 1-minute resistance exercise and returned to baseline after 15 minutes. Whereas, in the present study, reassessing immediately after exercise is not prioritized, this could have been the factor responsible for the noneffectiveness of the proposed exercise.

In human studies, the effect of exercise in relation to pain has also been observed. Hoffman et al. [21] evaluated whether patients with chronic low back pain would obtain analgesia induced by performing exercise on a stationary bike for 25 minutes. And the results showed analgesia in front of a pain stimulus, after the completion of the exercise, concluding that at least transiently applied exercise had a positive effect on pain.

Regarding the histological analysis of the sciatic nerve, it was observed that the injury model performed in this study was effective because it caused significant changes in the sciatic nerve of the animals. However, climb stairs resistance exercise on the proposed parameters failed to accelerate the healing process of all nerve fibers analyzed, but some fibers of the LEG showed up with normal features, like CG, showing a slight improvement of nervous tissue.

Corroborating this study, Raducan et al. [22] analyzed the histological characteristics of the sciatic nerve 24 hours, four days, two weeks, and four weeks after the injury of the nerve crush and found significant differences in nerve histology 24 hours after the lesion was not observed. However, the most obvious changes caused by axonal degeneration were observed after four days of injury and consisted of axonal swelling and disorganization of endoneurium, myelin degradation, and intense presence of phagocytic cells. However, at four weeks after lesion, most of the fibers were already regenerated, but with a thinner myelin sheath; however there were still some degenerate fibers, indicating that the regeneration process was incomplete. One can then see that possibly only 21 days which was the period of the present study has been little to analyze nerve regeneration, but the regenerative process was taking place, as it was possible to observe some regenerated nerve fibers in LEG.

On this same theme, Sta et al. [23] analyzed the relationship between electrophysiological, behavioral, and morphological parameters of the sciatic nerve in rats after a crushing injury and observed that the first signs of myelination begin from 21st PO, indicating that possibly it took a longer study to record significant data regarding the diameter of the myelin sheath.

Ilha et al. [24], among other variables, evaluated the effect of climb stairs resistance exercise, with an inclination of 80 degrees on Wistar rats sciatic nerve regeneration, starting after two weeks of injury and extending for 5 weeks after injury, using overhead that reached 250 grams at the end of treatment, and found that even with the stimulus of exercise regenerated fibers had thinner myelin sheath and larger spaces within the endoneurium.

Similar to this study, Teodori et al. [25] applied a resistance exercise protocol swimming in Wistar rats subjected to compressive lesion of the sciatic nerve and found that all the injured groups presented two to three times more axons than controls and explained that this is due the fact that, after nerve injury, each axon sprout generates various branches toward the target organs, increasing the number of axons and not reducing. They also noted that the DAX, DNF, and TMS in injured and treated animals were lower compared to the control (did only exercise) as well as in this study and explain that regenerated fibers with a small diameter are commonly found after peripheral nerve injury and are associated with withdrawal terminal connections during regeneration, and increased collagen, however with retraction of endoneurium. It is important to know that the animals studied were heavier and the kind of physical exercise was different, making it difficult to compare the data obtained.

In the study, Ilha et al. [24] also evaluated morphometric data, compared to the use of climb stairs resistance exercise on Wistar rats sciatic nerve regeneration and found that the group that underwent treatment with exercise had averaged DNF, DAX, and lower TMS to the control group, confirming what was found in this study. With respect to the G ratio, in this study, significant difference comparing CG with other groups was only observed, and CG showed closest average of 0.6. Unlike the present study, Ilha et al. [24] observed that the groups treated with climb stairs resistance exercise had high values of the ratio G, indicating delay in axonal regeneration and reduced nerve conduction velocity. In this study we used different physical exercise, like angle of the stairs and number of repetitions of exercises. Grinsell and Keating [2] reported that neurotrophic factors are present after the occurrence of nerve damage. Berchtold et al. [26] submitted Sprague-Dawley rats to perform exercise on the treadmill for 4 weeks and rated BDNF expression in the CNS of these animals. They found that exercise increased BDNF levels in the hippocampus of these animals compared to the control group.

As regards limitations of this study, it may be emphasized that the overload was similar in all the rats, not individualizing for each animal. And furthermore, evaluation of nociception occurred about 20 minutes after the exercise.

It is concluded from this study that in the proposed parameters the climb stairs resistance exercise was not effective in speeding the nerve regeneration process.

## Figures and Tables

**Table 1:** Data of mechanical allodynia measurements in different groups of study (mean ± standard deviation in grams) according to evaluation (baseline: BL; evaluation: EV).

Evaluations	Groups			
	CG	EG	LG	LEG
BL^•^	62.99 ± 15.69	81.33 ± 23.31	66.52 ± 6.33	70.04 ± 19.21
EV2	50.55 ± 8.21	59.45 ± 16.03	33.00 ± 11.75	28.25 ± 8.68
EV3	48.50 ± 13.10	52.45 ± 19.40	26.47 ± 9.36	29.95 ± 15.04
EV4^••^	42.94 ± 12.02	50.79 ± 10.29	27.95 ± 9.77	19.50 ± 5.85
EV5	52.05 ± 11.97	61.04 ± 10.75	22.09 ± 9.14	25.05 ± 9.60
EV6	59.50 ± 11.00	50.87 ± 15.17	31.28 ± 7.30	25.33 ± 7.01
EV7	49.44 ± 12.36	59.41 ± 6.49	45.57 ± 15.71	29.00 ± 15.63
EV8	45.94 ± 14.71	65.58 ± 10.21	51.71 ± 14.08	28.33 ± 7.01
Mean	51.48 ± 12.38	60.11 ± 13.95^*∗*^	38.07 ± 10.43^*∗∗*^	31.93 ± 11.32^*∗∗*^

^•^Significant difference compared to other evaluations; ^••^significant difference compared to EV7 and EV8; ^*∗*^significant difference compared to CG; ^*∗∗*^significant difference compared to CG and EG, *p* < 0.05.
